# Microdroplet Systems for Gene Transfer: From Fundamentals to Future Perspectives

**DOI:** 10.3390/mi16111245

**Published:** 2025-10-31

**Authors:** Mishell Criollo, Gina Layedra, Camilo Pérez-Sosa, Gustavo Rosero, Ana Belén Peñaherrera-Pazmiño

**Affiliations:** 1 Centro de Investigación Biomédica CENBIO, Facultad de Ciencias de la Salud Eugenio Espejo, Universidad UTE, Quito 170527, Ecuador; michucriollo01@gmail.com; 2 Special Coatings and Nanostructures Engineering (IREN), National Technological University, Buenos Aires 1706, Argentina; gina-layedra@hotmail.com (G.L.); camilo.bm91@gmail.com (C.P.-S.); gustavorosero@gmail.com (G.R.); 3 Facultad de Ciencias, Ingeniería y Construcción, Ingeniería Civil, Universidad UTE, Quito 170527, Ecuador

**Keywords:** microdroplets, conjugation, transformation, transfection, transduction, high-throughput, scalability, SG3, gene editing, CRISPR/Cas9

## Abstract

Microfluidics enables precise control of fluid movement within microchannels, facilitating the generation of microdroplets at high frequencies. This technology provides a unique platform for conducting biological and chemical experiments, enhancing throughput and sensitivity, particularly in single-cell analysis. The microdroplet environment enhances interactions between cells and gene delivery materials, resulting in greater contact area, higher reagent concentration, and improved diffusion for both eukaryotic and prokaryotic cells. This review discusses the advantages and limitations of transfection and transformation within microdroplet technologies, highlighting their potential to improve gene editing efficiency while addressing challenges related to delivery mechanisms and cellular uptake rates. The integration of microdroplet technology with advanced gene editing tools, such as CRISPR/Cas9, promises to streamline processes and improve outcomes in various applications, including therapeutic interventions, vaccine development, regenerative medicine, and personalized medicine. These advancements could lead to more precise targeting of genetic modifications, resulting in tailored therapies that better meet individual patient needs. Overall, the integration of gene delivery in microdroplets represents a significant leap in biotechnology, enhancing the efficacy of gene delivery systems and opening new avenues for research and development in precision medicine.

## 1. Introduction

The advent of microfluidics has facilitated the precise manipulation of fluids at the microscale level [1]. Microdroplet technology is an essential branch of microfluidics that yields droplets of exceptional monodispersity by utilizing two liquid phases mutually immiscible [2], specifically water/oil emulsions and liquid/gas interfaces. In droplet-based microfluidic frameworks, the generation of droplets between two fluids is controlled by the balance of inertial force, viscous force and interfacial tension force. The shear force and pressure gradient favor the breakup of a droplet under the action of the continuous phase, whereas the interfacial tension inhibits it [3]. Therefore, breakup occurs when the forces of shear and pressure exceed the stabilizing force of interfacial tension.

Reactions utilizing droplets in microfluidic systems provide superior accuracy and precision compared to traditional batch processes. Various factors significantly influence droplet-focused operations, including the choice of an ideal carrier fluid flow rate, the size of the droplets, and the quantity of droplets, all aimed at ensuring optimal reaction efficiency [4].

Indeed, droplet microfluidics provides a wide and practical toolset that facilitates the execution of chemical and biological investigations at accelerated rates and with enhanced efficacy relative to conventional instruments [5]. In fact, droplet microfluidics is one of the most successful microfluidic technologies which is transforming how chemists and biologists perform massive parallel experiments [6] and high-throughput screening [7]. The advantages provided by microfluidics pave the way for diverse opportunities to enhance the gene transfer parameters, leading to heightened transformation efficacy in prokaryotic [8] and improved transfection efficacy in eukaryotic cells [9] due to microdroplet exceptional features such as: large specific interfacial area and short diffusion paths [10], which together accelerate diffusion kinetics and augment mass transfer [1]. This environment not only increases reaction rates but also enhances the interaction efficiency between genetic material and cellular components.

The ability to transfer genetic material within microdroplets is the culmination of decades of research across two seemingly disparate fields: molecular biology and microfluidics. The biological foundation was laid first. The discovery of the “transforming principle” by Avery, MacLeod, and MacCarty who analyzed the transformation phenomenon of specific types of *Pneumococcus* [11] and the subsequent elucidation of DNA’s double helix structure by Watson and Crick [12] which unlocked the mystery of the genetic code by revealing how the genetic information is stored.

In parallel with these biological breakthroughs, a separate technological revolution in miniaturization was beginning. Early visions, like Lederberg’s oil chamber for isolating single cells [13], foreshadowed the coming shift. This trend accelerated with the development of the first “lab-on-a-chip” by Terry et al. [14] and the first droplet-based microfluidic experiment by Kawakatsu et al. [15].

As these two streams of innovation matured, scientists developed the critical techniques for gene manipulation that would eventually bridge the gap between them. Key milestones include the first successful DNA transfection [16], yeast transformation [17], and efficient RNA transfection methods [18].

**Figure 1:**
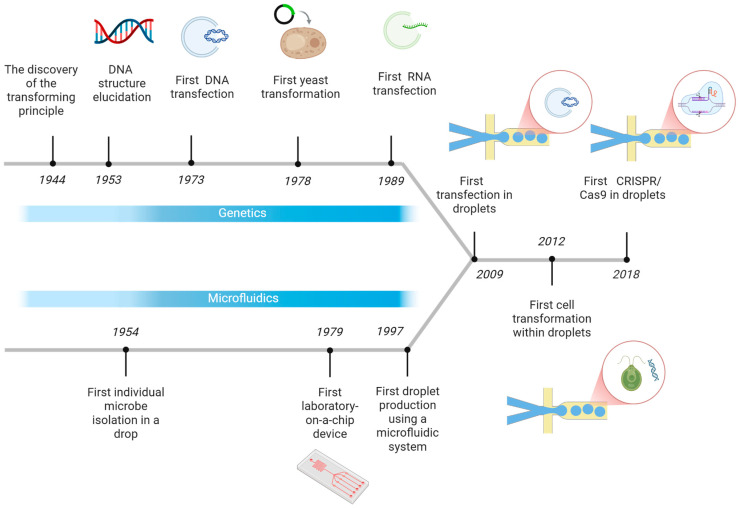
First steps towards gene transfer in microdroplets timeline. Created in https://BioRender.com accessed on 9 October 2025.

This convergence of foundational genetics, microfluidic technology (Figure 1), and established gene transfer protocols ultimately enabled the modern high-throughput analysis of genetic material in microdroplets. This powerful synergy is demonstrated by landmark achievements such as the first transfection of cells in droplets [19], the highly efficient transformation of *Chlamydomonas reinhardtii* [20], the recent integration of CRISPR/Cas9 systems for gene-editing in mammalian cells [21] and on-chip *Escherichia coli* transformation enabling electroporation multiplexing to improve industrial biosynthesis [22].

As already introduced, gene therapy offers the potential to transport genetic material into the target area, which occurs as dysfunction or disease at relevant tissue [23]. Nucleic acid elements like small interfering RNA (siRNA), short hairpin RNA (shRNA), micro RNA (miRNA), antisense oligonucleotides (ASO), plasmid DNA (pDNA), and CRISPR/Cas9 frameworks hold remarkable promise for therapeutic uses [24]. In essence, gene therapy represents a significant scientific frontier aimed at creating novel treatments and potential cures, thereby embodying the innovative spirit required to achieve the health and well-being Sustainable Goal 3 (SDG 3).

There exists a multitude of widely utilized and effective methodologies for the introduction of genetic information into microbial organisms, encompassing chemical competence, protoplasting, electroporation, and *E. coli*-mediated conjugation [8]. Confining individual microorganisms within droplets allows them to proliferate and generate metabolites faster than in a larger community found in 96-well plates or flasks [25]. In fact, the relation of microdroplet physical properties and plasmid transfer has been investigated at single-cell and individual droplet resolution [26].

Conventional methodologies for gene delivery, including lipotransfection and non-viral techniques, encounter significant challenges in addressing the limitations associated with suboptimal efficiency, elevated toxicity levels, immunogenicity, cell-type specificity and high material cost. Whilst membrane disruption methods, such as electroporation, penetration, nanoneedle injection and sonoporation result in cell damage and compromise viability [27,28].

In the context of innovative approaches, Single-Cell Transfection Technologies (SCTTs) have gained attention due to superior efficiency, safety and scalability than conventional transfection methods [29]. Additionally, coacervate microdroplet protocell-mediated gene transfection has been explored [30]. Besides, single-cell microfluidic chips have been validated to facilitate CRISPR/Cas9 DNA transfection, significantly enhancing the efficiency of transfecting hard-to-transfect cells [27]. Considering that various cell types possess unique optimal conditions for transfection, it is essential to devise a tailored and effective transfection technique by regulating the DNA or protein concentration within microdroplets. In the realm of high effective cell transfection, a microfluidic gene delivery platform called drop cell pincher (DCP) that enables gene deletion and insertion along with multiplexing has been reported [31]. This platform combines mechanoporation within microdroplets.

In this review we begin by outlining the use of microdroplets in gene transfer (cell conjugation, transformation and transfection). Next, we examine the hybrid and integrative approach to improve it by combining microdroplets with other techniques, followed by an overview of the applications of microdroplet technology in gene transfer across fields. We then explore the future perspectives highlighting contemporary droplet-microfluidic innovations that facilitate the miniaturization and automation of the gene-editing process.

## 2. Transformation in Microdroplets

### 2.1. Streptococcus Pneumoniae

Genetic transformation is a natural mechanism of horizontal gene transfer observed in several bacterial species. In *Streptococcus pneumoniae*, this process represents an adaptive strategy that enables the bacterium to respond to environmental stress. This process contributes to the emergence of more resistant strains, which can reduce the effectiveness of clinical treatments such as antibiotics and vaccines [32]. Transformation in *S. pneumonia*, a Gram-positive human pathogen, involves the uptake and integration of exogenous DNA released from other strains, usually following cell lysis. This occurs during a transient physiological state called competence, in which the bacteria express a specific set of genes that allow it to internalize extracellular DNA and recombine it into its own genome [33,34].

Due to its medical relevance and natural ability to develop genetic competence, *Streptococcus pneumoniae* has become an ideal model for studying transformation in microfluidic platforms, particularly in droplet-based systems. These systems allow the investigation of the transformation process at the single-cell level under highly controlled conditions. Traditionally, transformation studies in *S. pneumoniae* have relied on the addition of purified exogenous DNA to cultures of competent cells. However, this approach does not accurately replicate the natural mechanism, in which transformation occurs through direct contact between competent and non-competent cells, followed by lysis of the latter and immediate uptake of the released DNA [35,36].

Droplet-based systems enable the encapsulation of cell pairs within confined spaces, facilitating precise spatial and temporal control of the competence process. This setup makes it possible to study gene transfer at the exact moment of cell-to-cell interaction, providing a more representative model of the phenomenon as it occurs under natural conditions [34,35,36].

**Figure 2:**
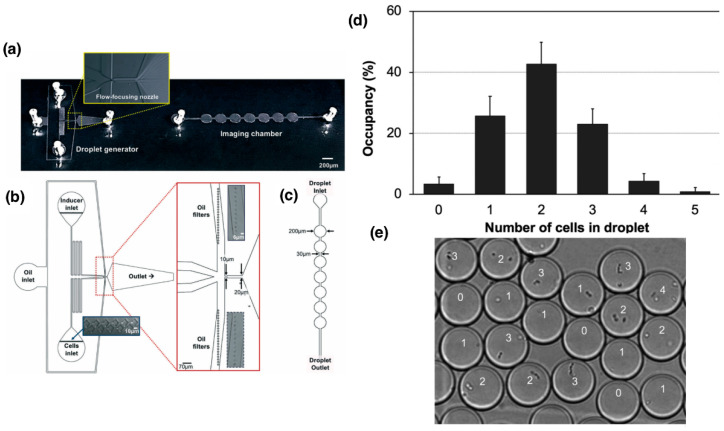
Use of microfluidic devices for droplet generation using *Streptococcus pneumoniae* as a model to study the transformation process. (**a**) General schematic of the device for droplet generation and the imaging chamber for cell counting analysis. (**b**) Detailed design of the device and the “T”-shaped junction for cell encapsulation and droplet formation. (**c**) Design of the imaging chamber optimized to facilitate counting and characterization of the formed droplets [35,37]. (**d**) Quantitative analysis of the number of cells encapsulated per droplet by manual counting. (**e**) Microscopic image of *S. pneumoniae* encapsulated cells in droplets of approximately 10 µm diameter. Numbers correspond to number of cells in each droplet. Adapted from reference [35]. License number: 1659531-1.

Lam et al. developed a microfluidic system designed with a “T”-shaped intersection where two flows were introduced (Figure 2): an aqueous phase containing *S. pneumoniae* bacteria and a continuous phase of fluorinated oil with surfactant. At the intersection, the oil flow sheared the aqueous flow, generating individual droplets that encapsulated one or more bacterial cells. This system allowed precise control over droplet size and generation frequency, as well as efficient encapsulation of cell pairs [34,37]. They used two bacterial strains: a competence-inducible recipient strain (CP2204, Rif^+^) and a non-competent donor strain (CP2215, NovR/SpcR). Competence was induced using a cocktail containing synthetic CSP (competence-stimulating peptide), BSA, and calcium ions. After incubation at 37 °C, competent cells lysed donor cells and took up the released DNA, acquiring resistance markers that were detected after droplet disruption and plating on selective media [35].

Encapsulation efficiency follows a Poisson distribution, as described by Lam et al. (2019) [35], which characterizes the random encapsulation of bacterial cells into droplets. This statistical model relates the probability of capturing a given number of cells per droplet to the average number of cells present in the aqueous suspension (λ), which depends on the initial cell concentration. By adjusting λ through dilution, it is possible to modulate the frequency of single-cell or paired-cell encapsulation events. In their experiments, most droplets contained one or two *Streptococcus pneumoniae* cells, consistent with the Poisson distribution expected for random encapsulation during droplet formation, an arrangement ideal for studying direct cell-to-cell transformation events. Transformation inside the droplets produced recombinants at rates comparable to those observed in planktonic cultures, demonstrating that competence development and DNA uptake remained functional in the confined environment. When dilution after droplet disruption was minimized (1:10), transformation could continue outside the droplets; however, a higher dilution (1:1000) effectively suppressed this subsequent transformation, confirming that gene transfer primarily occurred within the droplets [35].

The results reported on the transformation process within microdroplets evaluated both cell viability after recovery and transformation frequency, by detecting how many cells acquired resistance genes. Although cell viability was lower in the droplets compared to the non-encapsulated controls, the transformation frequency was comparable. These findings validated that confinement in microdroplets does not limit cell-to-cell genetic transfer [35].

Genomic analysis of 88 recombinants showed that DNA transfer was not limited to the selected marker, but that on average each clone incorporated three additional blocks of genetic material in different chromosomal regions. The transferred fragments ranged from 858 bp to 89 kbp, which in some cases represented up to 4% of the total *S. pneumoniae* genome. These results showed that microdroplets reproduce not only classical transformation but also macro-recombination, a phenomenon previously associated with biofilms and in vivo conditions. Thus, the work of Lam, Brennan et al., 2019 [35] complements that of Lam, Maienschein-Cline et al., 2019 [37] by showing that, beyond maintaining efficiency levels similar to liquid cultures, confinement in droplets allows us to unravel the magnitude and complexity of the genetic exchanges that characterize this pathogen.

### 2.2. Algae

The development of microfluidic systems has incorporated microalgae as biological models, with *Chlamydomonas reinhardtii* being one of the most widely used due to its rapid growth, ease of cultivation, and extensive genetic characterization, making it a standard organism for study. In genetic engineering, a major challenge has been overcoming the barrier imposed by the cell wall and plasma membrane, which limits the uptake of exogenous genetic material. Electroporation (explored in greater depth in Section 4.2) has therefore been employed as a transformation method. The transformation of *Chlamydomonas reinhardtii* was conducted in microdroplets and the transformation efficiency via microfluidic electroporation was shown to be more than three orders of magnitude higher for the wall-less mutant and more than two orders of magnitude higher for the wild type than bulk phase electroporation [20].

## 3. Transfection in Microdroplets

Following the advances achieved in applying microdroplet-based microfluidic systems to unicellular models, the approach has been extended to eukaryotic cells with the aim of optimizing genetic delivery processes. In this context, droplet microfluidic platforms have been adapted to facilitate non-viral and viral transfection in mammalian cells, enabling the exploration of their potential for controlled gene transfer and the development of tools for therapeutic applications.

### 3.1. Non-Viral Transfection

Droplet-based microfluidic platforms provide an advantageous environment for transfection and gene editing in eukaryotic cells. The encapsulation of single cells within picolitre volumes enhances contact between nucleic acids, proteins, or complexes and the cellular membrane, while simultaneously reducing reagent consumption and cytotoxic effects. In contrast to conventional methods such as lipofection, electroporation, or viral delivery, the droplet format offers a more controlled and homogeneous microenvironment, favoring precise modulation of reaction kinetics. The possibility of working at single-cell resolution, combined with the scalability and automation inherent to microfluidic technologies, has positioned microdroplets as a promising tool for advancing stem cell engineering and other demanding biotechnological applications.

Evidence from recent studies illustrates how droplet microfluidics can improve established non-viral delivery strategies. In the case of transposase-mediated transfection of human-induced pluripotent stem cells (hiPSCs), the use of PiggyBac within microdroplets resulted in efficiencies that exceeded conventional plate-based controls. The confinement of cells together with transposase and donor DNA within droplets increased the probability of stable genomic integration, yielding more reliable and homogeneous cell populations. These findings highlight how physical confinement not only reduces variability but also accelerates the generation of engineered stem cell lines suitable for both research and therapeutic purposes.

**Figure 3:**
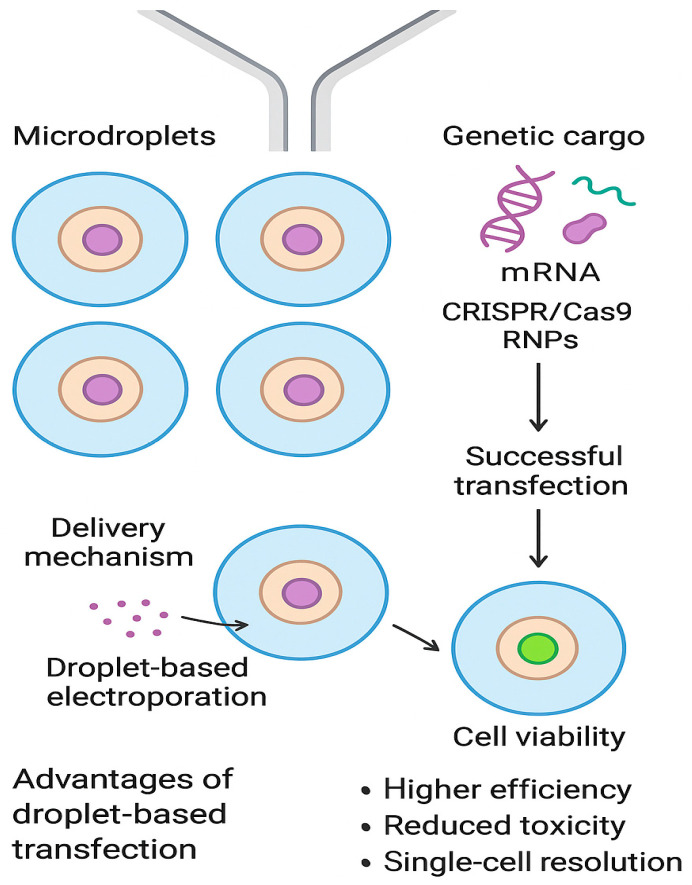
Schematic illustration of microfluidic droplet-based single-cell transfection. Created in DALL-E.

Comparable advances have been observed in genome editing using CRISPR/Cas9. Although overall editing efficiency within droplets may remain similar to that of bulk culture methods, the microdroplet format provides unique advantages in the subsequent isolation and expansion of clones. When hiPSCs were encapsulated in droplets supplemented with Geltrex hydrogel, single-cell capture (Figure 3) and survival were markedly improved. This strategy enabled the recovery of edited clones without antibiotic selection, thereby simplifying workflows and improving clonal fidelity. In regenerative medicine and disease modeling, where the precise derivation of isogenic cell lines is essential, such improvements represent a significant step forward [38].

Recent advances in droplet-based and hydrogel-assisted transfection systems have demonstrated the potential of alginate microgels as efficient, biocompatible carriers for nonviral gene delivery. In particular, ref. [39] developed a layer-by-layer biomimetic microgel platform composed of alginate and chitosan, which enabled single-cell encapsulation and localized transfection within 3D microenvironments. The alginate microdroplets provided mechanical protection and controlled release of genetic cargo, while maintaining high cell viability (>85%) and moderate-to-high transfection efficiencies (ranging from 40–60% depending on cargo and coating composition). The system was also compatible with high-throughput microfluidic production, underscoring its potential for scalable single-cell transfection and nonviral gene therapy applications.

### 3.2. Viral Transfection

Viral based transfection, or more known as transduction, is a way to transfer genetic material using a viral vector into a host cell [40]. There are many viral vector systems that are currently used for gene delivery in immune cells like lentiviruses, adenovirus, and adeno associated viruses, gamma retroviruses [41]

Viral-based systems remain a widely used strategy for delivering exogenous genetic material into mammalian cells; however, their preparation for genome-editing applications is labor-intensive [42,43]. To overcome this limitation, a microfluidic platform was adapted to integrate viral generation, packaging, and transduction into a single device. The key innovation is the reduction of reaction volume, which accelerates transduction and increases overall efficiency. This platform also enables the evaluation of RNAi or CRISPR/Cas9 systems, while shortening the time required for lentiviral production and transduction from several weeks to only a few days [42].

**Figure 4:**
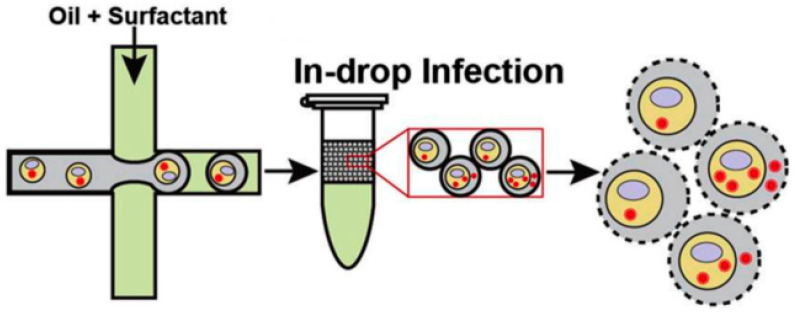
Virus transfection using microdroplets. Infected cell suspensions were encapsulated into droplets within a fluorinated oil continuous phase and incubated for 24 h at 37 °C under 5% CO_2_. The final stage illustrates cell lysis, which was used to analyze viral production [44]. Used under Creative common license.

Complementally, drop-based microfluidics has been applied to study virus–host interactions at the single-cell level by encapsulating cell lines (A549, MDCK, siat7e) in droplets, facilitating viral propagation in isolated microenvironments. This approach supports adherent cell culture without suspension adaptation and also provides a platform to investigate viral replication, transmission, and virulence across variants (Figure 4) [44].

## 4. Hybrid and Integrative Approach

Although droplet-based systems enhance the efficiency of direct genetic material delivery compared to conventional techniques, optimizing outcomes remains challenging, particularly in recalcitrant or hard-to-transfect cells, in both unicellular and multicellular organisms. In this context, integrating strategies that facilitate membrane permeabilization while minimizing cellular damage and preserving viability is essential to improve the performance of existing platforms and to establish a foundation for their scalability in genetic editing applications.

### 4.1. Cell Mechanoporation

The mechanoporation strategy employs mechanical forces to induce transient nanopores in the cellular membrane, enabling the delivery of biomolecules into the cytoplasm. By eliminating the use of external energy carriers, this approach minimizes cell toxicity and structural damage [45].

**Figure 5:**
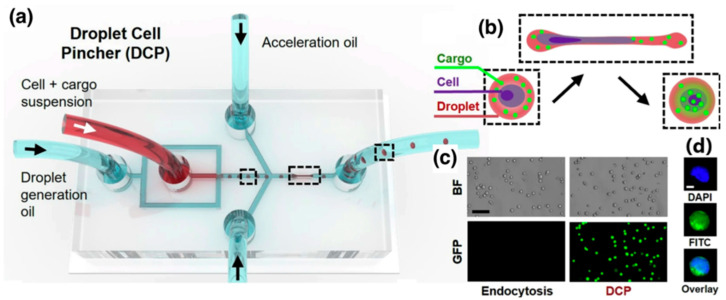
Design and fundamental operating principle of the DCP platform, which integrates microfluidics with cell mechanoporation. The figure shows how the device uses (**a**,**b**) flow-focusing geometry to (**c**,**d**) encapsulate cells and the target CRISPR-Cas9 system’s ribonucleoprotein (RNP) into uniform droplets. The dotted line highlights the droplets inside the device. Crucially, it demonstrates the mechanoporation process: these droplets are accelerated and forced to pass rapidly through a single, narrow microscale constriction. This controlled mechanical squeezing physically stresses the cells, inducing the formation of transient discontinuities (pores) in both the cell membrane and the nuclear membrane. This permeabilization allows for the rapid, convective internalization of the genetic material directly into the nucleus, highlighting the core mechanism responsible for the platform’s high efficiency and success in genome editing [31]. Used under Creative Commons Attribution (CC BY) license.

The Droplet Cell Pincher (DCP) platform exemplifies a highly controlled form of a physical permeabilization. In this system, cells and CRISPR components are co-encapsulated within uniform microfluidic droplets. These droplets are then rapidly accelerated and precisely guided through a single, microscale constriction within the chip. This high-speed, mechanical compression induces significant but temporary stress, leading to the formation of transient discontinuities (pores) in both the cell membrane and, crucially, the nuclear membrane. This dual-membrane perforation facilitates the rapid, convective internalization of the CRISPR-Cas9 RNPs directly into the nucleus (Figure 5).

This convective process is a major advantage over older microfluidic methods that rely solely on passive diffusion, which often fails to penetrate the nuclear envelope and thus results in suboptimal editing efficiency, particularly for complex tasks like gene knock-in. The DCP platform demonstrates remarkable success and versatility in its applications, proving its capability to deliver various macromolecules, including messenger RNAs (mRNAs, with ~98% success) and plasmid DNAs (pDNAs, with ~91% success). More importantly, in comparative genome editing experiments, the DCP consistently and significantly outperformed electroporation—the current state-of-the-art method. Specifically, DCP achieved editing efficiencies that were approximately 6.5-fold higher for single gene knockouts and 3.8-fold higher for both double knockouts and gene knock-ins (via homology-directed repair, HDR).

Beyond superior efficacy, the mechanoporation approach provides a critical safety benefit: it causes less physical stress and induces fewer morphological changes in the treated cells compared to the high-voltage electrical pulses used in electroporation. These findings firmly establish the DCP platform as a more efficient, versatile, and genetically stable next-generation tool with powerful implications for both clinical cell-based therapies and fundamental biological research [31].

### 4.2. Electroporation

This is a technique that uses short electric pulses to temporarily create pores in cell membranes [46]. The electroporation occurs when the cell-containing droplets (in oil) flow through a pair of microelectrodes with a constant voltage established in between.

**Figure 6:**
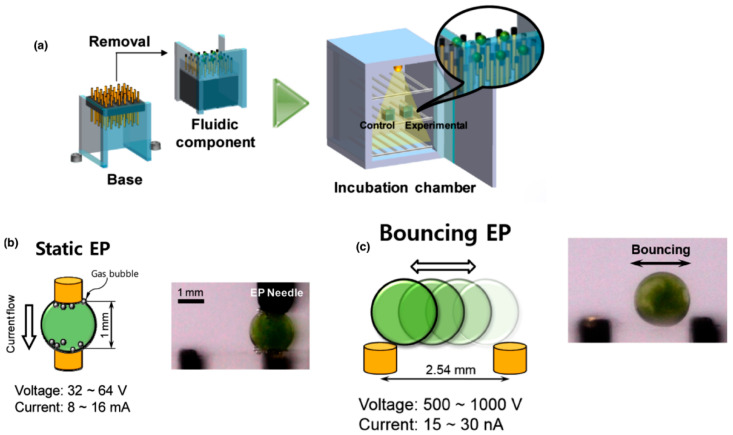
Schematic representation of the digital microfluidic electroporation system developed by Im et al. (2015). (**a**) Fabrication and assembly of the chip using an electrode array encapsulated in PDMS connected to a removable base and enclosed within an incubation chamber, allowing stable droplet manipulation. (**b**) Static droplet electroporation. (**c**) Bouncing droplet electroporation. Both modes enable efficient transformation of *Chlamydomonas reinhardtii.* Adapted from [47]. License number: 6137320641251.

In 2015, Im et al. [47] developed a digital microfluidic electroporation system in which droplets served as independent microreactors encapsulating *C. reinhardtii* cells with plasmid DNA (Figure 6). Two droplet-based modes were tested: static droplet EP, where the droplet remained fixed between electrodes and achieved up to 21% transgene expression efficiency, and bouncing droplet EP, where droplets oscillated between electrodes, showing lower efficiency but reduced contamination risk and potential for on-chip culture. Both approaches significantly outperformed conventional bulk electroporation [47].

Subsequently, ref. [48] optimized this system by introducing fluorescent indicators (Yo-Pro-1) to rapidly monitor DNA uptake and by fine-tuning critical parameters such as temperature, voltage, pulse number, and cell concentration. These improvements not only preserved cell viability but also enabled the generation of stable, antibiotic-resistant transformants maintained for months, demonstrating the feasibility of this microfluidic platform for long-term genetic transformation in microalgae.

The system’s technological foundation is an innovative droplet microfluidic chip featuring a 10 × 10 array, enabling 100 independent genetic modification reactions in parallel. Automation is achieved through seamless integration with commercial liquid-handling robots, leveraging a standard 384-well plate template for sample input. Central to the device’s function are two electric field-actuated operations within each isolated droplet: electrowetting, which controls surface tension to achieve on-demand mixing of reagents, and electroporation, which mediates the transformation of cells with the CRISPR payload. This integrated, contactless approach eliminates the need for manual reagent premixing and bulk processing, boosting efficiency while preserving precious reagents.

**Figure 7:**
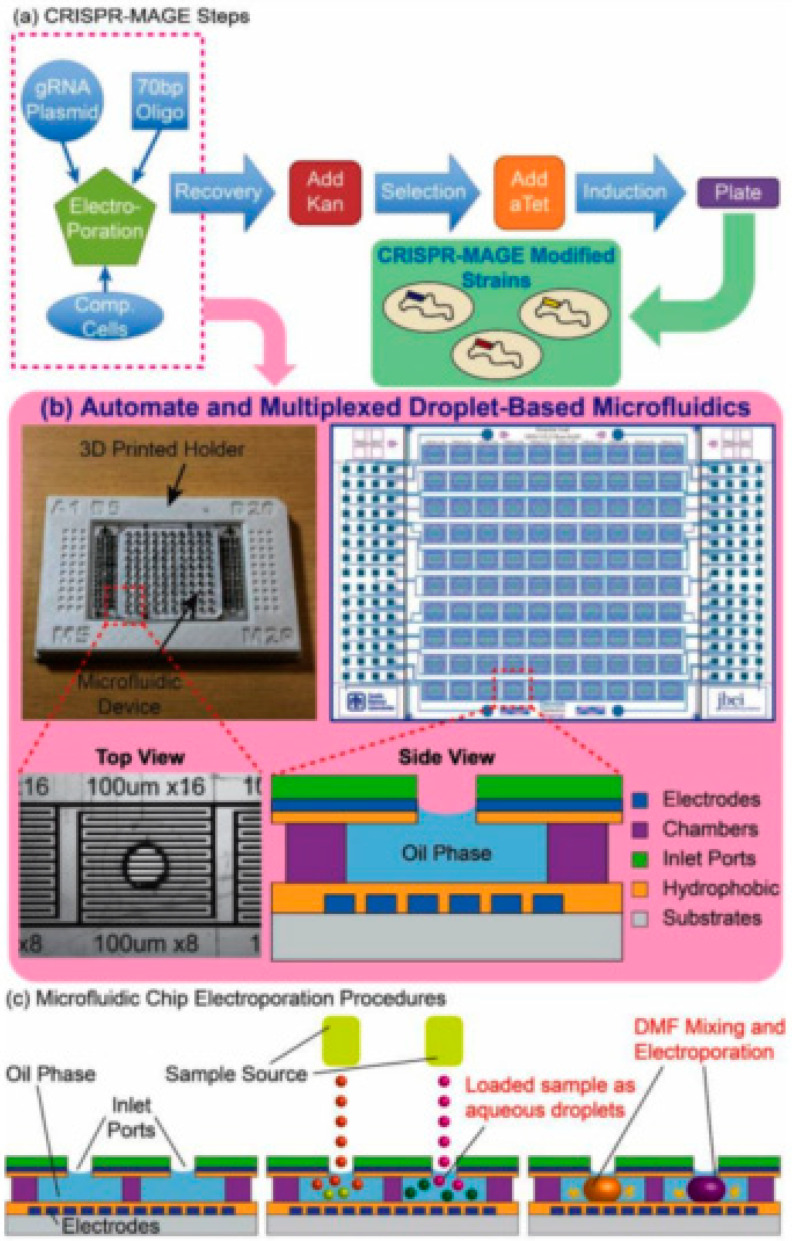
(**a**) This figure provides a comprehensive overview of the automated CRISPR-MAGE workflow and the microfluidic hardware that enables it. The physical device is a highly scalable platform, featuring 100 discrete reaction chambers with individually addressable electrodes, designed to conform to the 384-well plate format for seamless integration with commercial lab automation equipment. (**b**) The microfluidic chip in a 3D printed holder (**left**), the electrode pattern (**right**), a top-view of an individual well, and a side-view schematic of a well. The chip is designed to contain 100 discrete reaction chambers with individually addressable electrodes for multiplexed CRISPR-MAGE recombineering, and its 384-well format design can be interfaced with lab automation equipment. (**c**) Droplets containing plasmids and cells are dispensed into each chamber through the inlet port, mixed by electrowetting, and electroporated by applying a voltage pulse [37]. Used under Creative Commons Attribution (CC BY) license.

The platform’s high efficacy was validated using CRISPR-MAGE (Multiplex Automated Genome Engineering) (Figure 7) to perform targeted genomic modifications in *E. coli*. The authors demonstrated successful engineering across two critical test cases: validating the system’s function via the disruption of the galK gene and, more significantly, performing combinatorial engineering of glnA and bpsA to enhance indigoidine production. These results underscore the chip’s robust ability to execute high throughput, multiplexed genetic modifications necessary for strain optimization. By enabling rapid and parallelized testing of numerous genetic variants, the platform drastically accelerates the critical Design–Build–Test–Learn cycle in metabolic engineering [37].

### 4.3. Ultrasonic Levitation

**Figure 8:**
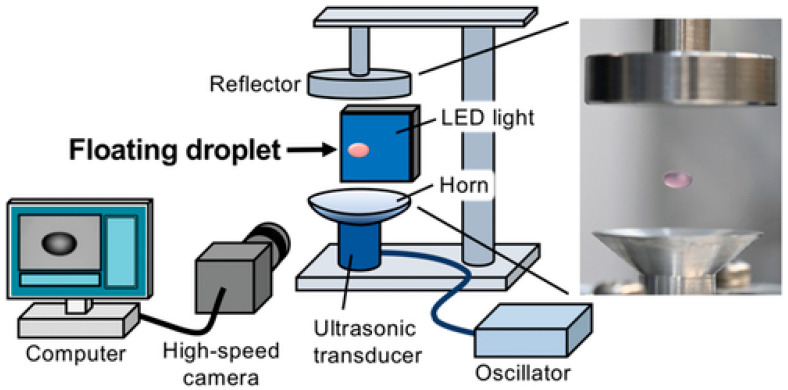
The figure illustrates the suspended droplet, often shown containing a cell and pDNA complex suspension. The entire system is integrated with a high-speed camera and an associated measurement system. It allows the droplet’s dynamics and accurately calculates its volume in real-time [49]. Used under Creative Commons Attribution (CC BY) license.

Methodology for cell engineering by demonstrating successful transfection of animal cells within a floating droplet (Figure 8). The study directly addresses the critical environmental issue of massive plastic waste generated in life science laboratories. By utilizing ultrasonic levitation, the technology enables researchers to conduct chemical and biological reactions in a substrate-free environment, circumventing the need for conventional plastic consumables like culture plates and vials, thus forming the basis of a truly sustainable technology platform.

The core of the technology involves suspending a droplet of cell culture medium, containing both the animal cells (e.g., Huh-7 cells) and the genetic material (e.g., plasmid DNA), using single-axis acoustic levitation generated by ultrasonic standing waves. This mechanism allows for a fully contactless reaction, which is a major advantage for maintaining sample purity and reducing contamination risks, as the sample never touches a solid surface. Furthermore, the levitation process was found to significantly alter cell characteristics, specifically consolidating the normal endocytic uptake mechanism into macropinocytosis. This change in the cellular uptake pathway is hypothesized to be the key factor driving the remarkably high efficiency of genetic material internalization [49].

### 4.4. Voltage-Driven Digital Microfluidics

Digital Microfluidics (DMF). The system addresses a critical need in cell engineering for platforms that can handle small, precious cell samples (as few as ≈40,000 cells) and complex biological payloads (like Cas9 RNPs, plasmids, and mRNA) while minimizing cellular stress and reagent consumption [50].

**Figure 9:**
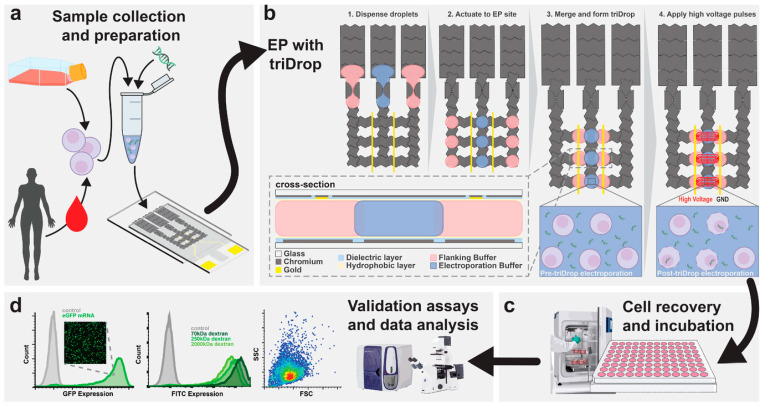
The figure shows (**a**) cell sample was cultured and prepared by resuspending it in an electroporation buffer with target delivery molecules (**b**) The schematic illustration shows top view of the DMF device and the formation of the TriDrop through a series of electrode actuations. The red lines (in frame 4) indicate the electric field lines generated during application of high-voltage pulses. The in-set shows the cross-sectional view of the triDrop. (**c**) After electroporation, samples were then incubated (>24 h) for cell recovery and followed by (**d**) analysis using FACS or with fluorescent microscopy or any analytical method of interest. License Number: 6137321364871 [50].

The success of the triDrop hinges on the precise control afforded by voltage-driven digital microfluidics. DMF operates by manipulating discrete microdroplets on a planar electrode array using electrowetting-on-dielectric (EWOD) principles, where applying voltage causes droplet movement, merging, and splitting (Figure 9). In the triDrop architecture, the system is designed as a “liquid cuvette” formed by merging three distinct droplets: two outer droplets composed of high-conductivity liquid that contact the electrodes, sandwiching an inner droplet of low-conductivity liquid that contains the cells and the genetic cargo [50].

The delivery mechanism—electroporation—is specifically enabled and optimized by the strategically applied voltage across this tri-droplet structure. By applying voltage to the conductive outer droplets, a focused and effective electric field is generated across the central, low-conductivity droplet. This field is precisely tuned to induce transient pore formation in the cell membranes, allowing the genetic cargo to be inserted. By isolating the cells in the low-conductivity middle droplet, they are shielded from the high current density and direct contact with the electrodes and their harmful byproducts, ensuring efficient payload insertion without sacrificing long-term cell health or functional viability [50].

### 4.5. Acoustic Droplet Ejection Technology

The methodology focused on the simultaneous optimization of the three critical parameters for a successful transfection protocol: the dilution of the transfection reagent, the amount of DNA, and the starting DNA concentration. Experiments were conducted in 384-well plates, leveraging the high throughput and precision of acoustic droplet ejection. The authors defined a four-step protocol for transfecting HeLa cells, which included the contactless dispensing of nanoliter quantities of plasmid DNA and transfection reagent. This miniaturized approach not only conserved reagents but also allowed for the rapid identification of optimal assay conditions through automated screening.

The main findings demonstrated the high efficacy and reliability of the developed protocol. The optimal settings defined through the screening allowed the authors to achieve transfection efficiencies of up to 90% in HeLa cells. Furthermore, in co-transfection experiments (introducing two different plasmids), the protocol achieved a co-expression of nearly 100% within the transfected cells, a significant achievement that validates the system’s precision in dispensing minute volumes of multiple reagents. These results confirm that acoustic droplet ejection technology is a viable, fast, and automated alternative to manual or low-throughput transfection methods [51].

### 4.6. Acoustophoresis

**Figure 10:**
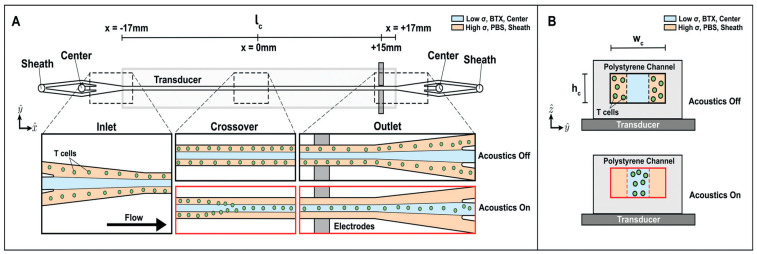
Acoustophoretic media exchange and electrotransfection microfluidic device. T cells in high conductivity media are introduced into the device sheath inlet, and low conductivity BTX electroporation buffer is introduced into the center inlet. Flow travels down the length (l_c_) of the channel, designated in the $\hat{x}$-direction. The width (Wc) is in the $\hat{y}$-direction, and the height (hc) is in the $\hat{z}$-direction. (**A**) Illustration of top-down view of channel with enlarged views of the inlet, crossover region, and outlet. The microchannel is mounted on top of a transducer. When an acoustic actuation is applied to excite the channel, cells in the sheath will be driven to the center of the channel, represented as “Acoustics On”. The T cells cross over from the sheath stream into the center upstream of the midpoint of the channel, and the T cells are transported into the center stream by the time they flow past the electrode region toward the end of the channel. If acoustics are off, the cells remain in the sheath and are collected in the sheath outlet. (**B**) Illustration of the cross-sectional view of the device channel mounted on a transducer showing where the T cells are located when acoustics are turned off and on [52]. Used under Creative Commons Attribution (CC BY) license.

The system’s operation is based on the precise control afforded by acoustophoresis within a continuous laminar sheath flow (Figure 10). The microfluidic channel is fabricated from polystyrene for low-cost, disposable clinical use and is mounted on a piezoelectric transducer to generate ultrasonic standing waves. T cells, initially suspended in high-conductivity culture media, enter the side streams and are acoustophoretically transported toward the pressure node at the channel’s center. This center stream carries the low-conductivity electroporation buffer and the mRNA payload. As the acoustically focused cells reach the channel’s end, they pass between integrated platinum electrodes, which deliver the pulsed electric fields for transfection. This hydrodynamic configuration protects the cells from the intense field regions and cytotoxic byproducts at the electrode surfaces.

Experimental results validated the system’s dual functionality and efficiency with primary human T cells. The acoustic field achieved a media exchange efficiency of 87.5%, successfully moving cells from the sheath stream into the electroporation buffer. This transfer occurred in seconds, significantly minimizing cell exposure to the potentially harmful buffer. Furthermore, the system demonstrated successful transfection of mCherry-encoding mRNA, with efficiencies increasing up to 60% with increasing voltage. Crucially, throughout all tested conditions, the controlled, rapid process maintained high cell health, resulting in less than a 5% reduction in viability. No transfection was observed without acoustic stimulation, proving the precision of the cell localization mechanism [52].

## 5. Applications Across Fields

**Figure 11:**
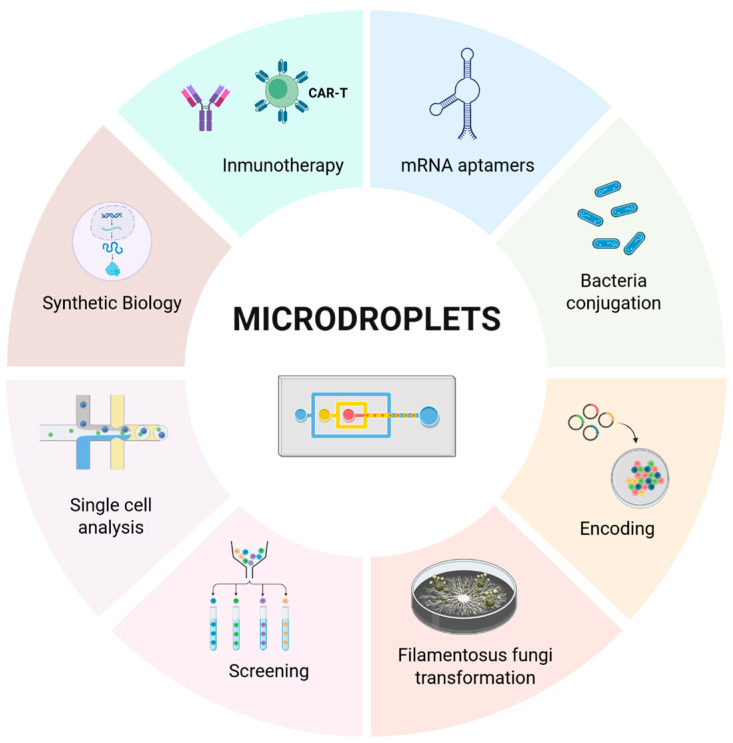
Microdroplets for gene delivery and other applications. Created in https://BioRender.com accessed on 9 October 2025.

In biological and biomedical research, microfluidic systems represent a promising technology owing to their ability to operate with minimal volumes of reagents and biological samples. This characteristic leads to reduced reagent costs, shorter analyzing times, and improved efficiency in processes such as gene delivery. In particular, microdroplet-based platforms constitute a powerful approach (Figure 11), as they enable precise control over droplet size, facilitate efficient mixing of solutions, and significantly minimize the risk of cross-contamination [23].

### 5.1. Synthetic Biology

Microfluidic technologies are transforming synthetic biology by addressing the limitations of traditional experimental approaches. Compared with manual and robotic methods, they provide higher throughput, greater reproducibility, reduced reagent consumption, and improved process control [53,54]. These advantages have accelerated key applications such as DNA assembly, gene delivery, cell culture, phenotypic assays, and the construction of genetic circuits [53,55]. More recently, droplet microfluidics has expanded these capabilities, enabling precise and reproducible encapsulation of functional biological components, biomolecules and nucleic acids, offering a powerful approach for artificial cell production [56,57].

Droplet-based systems have been applied for the encapsulation of artificial cells and coacervate protocells, the study of membrane properties, and the high-throughput synthesis of lipid vesicles [56,58,59]. In biomedical applications, membrane-free coacervate microdroplet protocells combined with plasmids have been used as synthetic cargo carriers to enhance transfection. This approach enabled nitric oxide synthase gene delivery in the SMMC-7721 cancer cell line, improving expression of this signaling molecule, which plays key roles in immune responses, apoptosis, and cancer [30].

Also, [60] further employed this approach to generate droplets containing beads coated with DNA encoding different riboswitches for incorporation into cell-free systems. This method enables efficient screening by ensuring that most droplets carry only a single riboswitch variant, as verified through fluorescence quantification. This advancement is particularly significant, as riboswitches function as both chemical detectors and genetic regulators without the need for protein factors [61].

### 5.2. Immunology and Gene Therapy

In personalized immunotherapy, chimeric antigen receptor (CAR) T-cell therapy has emerged as a cornerstone of cancer treatment, requiring the generation of approximately 10^8^–10^9^ engineered lymphocytes for a single infusion [62]. In the same way, mRNA administration has emerged as a potential technology that enables the delivery of genome-editing molecules and the transient expression of specific proteins in immune cells, while eliminating the risk of insertional mutagenesis. Nevertheless, in ex vivo therapeutic approaches, mRNA transfection into lymphocytes remains challenging, as cell viability is frequently compromised by the cytotoxic effects of electric fields. These adverse effects are inherent to electroporation, which, despite being the predominant technique employed for this purpose, often reduce the functional recovery of the transfected cells [63].

**Figure 12:**
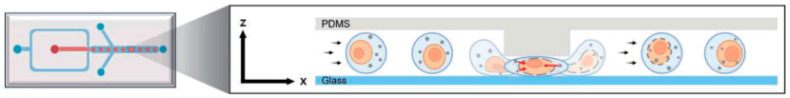
Schematic representation depicting the stepwise z-axis droplet squeezing intracellular delivery mechanism [64]. The figure illustrates the rapid deformation of the cell and the transient formation of pores that enable cargo entry (red arrows), followed by subsequent recovery of the cell membrane (black arrows). License number: 1659928-1.

To overcome this weakness, diverse platforms have been developed; for instance, ref. [62] reported a microfluidic intracellular delivery system based on mechanical squeezing, which significantly improved gene transfer, cell recovery, and process scalability in T lymphocytes (Table 1). The mechanical compression of the cell membrane allows the reception and the internalization of gene material, even at low concentrations. Also, ref. [64] reported that the combination of diffusive and connective flow enables efficient mRNA transfection. The high delivery efficiency is attributed to rapid cell deformation and restoration (Figure 12). In this context, these platforms demonstrate versatility and cost-effectiveness, while also facilitating the delivery of various functional nanomaterials and genome editing systems including mRNA, which is critical for the advancement and success of cell-based therapies.

In immune cell engineering, droplet-based microfluidic systems have emerged as a powerful platform for the controlled and cost-effective delivery of gene-editing components across several cell models. Also, their capacity to encapsulate complex cargo, such as Cas9 ribonucleoprotein complex, and reduce high cell death rate make them particularly valuable for application in the therapeutic field [50]. In primary human T cells, these systems enable efficient genome editing, achieving modification rates of up to 80% (Table 1). The compartmentalization of individual cells within droplets facilitates localized exposure to CRISPR/Cas9 reagents, thereby maintaining high viability while minimizing off-target effects.

The versatility of this approach has also been demonstrated in adherent and suspension cell lines relevant to biomedical research. In HEK 293T cells, a high-throughput droplet-based single-cell transfection platform has been used to achieve targeted CRISPR/Cas9 knockouts through encapsulation of DNA–transfection reagent complexes, which enhance intracellular delivery and transfection efficiency [27]. Similarly, lipoplex-mediated transfection within droplets has been shown to substantially increase delivery efficiency in suspension cell lines such as K562 and Jurkat compared with conventional bulk techniques, while preserving cell integrity [65].

Beyond immune cell engineering, droplet microfluidics is increasingly applied in oncology. For instance, the Automated CRISPR Editing (ACE) platform integrates cell culture, gene editing, and image-based analysis within microfluidic channels for the study of lung cancer models. Using CRISPR/Cas9 delivered via plasmids and lipid vesicle complexes, this system enables precise gene knockouts and enhances the intracellular transport of exogenous material through endocytic pathways [21].

Moreover, recent studies have highlighted the utility of microfluidic systems in supporting the development of next-generation immunotherapies. In particular, the integration of an autocrine-based lentiviral transduction system with droplet microfluidics has been employed to overcome key limitations in the evaluation of bispecific and agonist antibodies, including restricted diversity, low throughput, and screening bias. In this approach, single infected cells were co-encapsulated with receptor cells into a droplet, to improve both antibody secretion and the efficiency of transduced cell screening [66].

### 5.3. Other Microdroplet Contributions

- Microdroplet platforms for Bacterial Conjugation

Efficient DNA delivery remains a major challenge in synthetic biology. Conventional methods, such as electroporation and chemical competence, are effective mainly in model microorganisms but are less applicable for non-domestic strains. Microfluidic platforms address this limitation by using microdroplets as bioreactors, where donor and recipient cells are brought into controlled contact. Ref. [67] evaluated this system and demonstrated that it enhances DNA transfer through conjugation and expands the range of host species available to be a chassis of genetic engineering, opening new possibilities for synthetic biology applications. Also, this methodology has been automatized through XPORT ENTRAP device, which increase horizontal transfer genes [8].

- Microdroplet-based workflow for filamentous fungi transformation

Filamentous fungi have been chosen as production hosts for recombinant proteins due to their capacity to produce eukaryotic proteins [68]. Albeit strain improvements are needed to achieve higher production yields. Therefore, genetic transformation techniques have enabled the generation of hyper-productive strains [25]. In this context, Luu et al. developed a high-throughput workflow using a droplet-driven microfluidic platform to genetically modify filamentous fungi [25].

- Microdroplets for screening of protoplast

Microdroplet systems have been applied in plant research to address limitations in phenotypic analysis, such as low throughput and time-consuming procedures. Using *Marchantia polymorpha* as a model, the authors encapsulated single transformed protoplasts within droplets to quantify chlorophyll content and fluorescence intensity. This platform enables efficient assessment of gene expression activity and provides a cost-effective approach for screening transgenic plants. Additionally, it allows the evaluation of environmental responses as well as the functionality of genetic circuit components [69].

- Droplet-on-demand microfluidics for single-cell catalysis

Yeast-catalysed transformation from ketoester to ethyl-3-hydroxybutyrate reaction in droplets has been monitored by coupling of active droplet generation to electrospray ionization/mass spectrometric detection (ESI/MS) [7]. As association of droplets with the MS signal in high-throughput droplet analysis is challenging, authors circumvent this problem by using a droplet-on demand system for droplet generation by hydrodynamic gating with downstream microscopic droplet detection and MS analysis. They ensured that each droplet can be detected in the MS by using 1 Hz to achieve good droplet spacing. Besides, they examined droplets with two microscopes, one for horizontal observation and a portable microscope for lateral observation due to some yeast cells settling at the bottom of the droplet after a few seconds.

- Droplets for single cell isolation

Microdroplet systems can be integrated with digital microfluidics (DMF) to enhance the recovery of gene-edited single cells from lung carcinoma populations. Encapsulation enables isoclonal sorting of heterogeneous populations carrying knockout mutations while simultaneously improving cell viability. Furthermore, this approach can be extended to other cell types, including primary cells and stem cells, thereby broadening its applicability in cellular engineering and biomedical fields [70].

- Droplets for single cell—cell interaction

In a subsequent study, Lam et al. [37] expanded the microfluidic approach by sequencing the complete genomes of the recombinants obtained in droplets. Their objective was to compare the results of confined crosses with those of parallel liquid cultures, evaluating whether microdroplets could faithfully reproduce the dynamics of natural transformation. The authors observed that transformation frequencies were comparable in both contexts, with a slight reduction attributed to the presence of single-cell droplets and the effect of the surfactants used, although these differences were not statistically significant. These findings confirmed that confinement does not compromise the efficiency of the process, validating microdroplets as a robust model for studying cell-to-cell interactions.

- Droplet encoding

In a very recent work, Shang et al. have developed a platform for multiplex foodborne pathogen detection, where *Salmonella typhimurium*, *E. coli*, *Listeria monocytogenes*, *Pseudomonas aeruginosa*, *Staphylococcus aureus*, *Vibrio parahaemolyticus*, and *Bacillus cereus* were main target objects. Harnessing the signal amplification of CRISPR/Cas13a system and droplet-based digitization made possible ultrasensitive detection of target bacterial nucleic acid and heightened detection throughput. This one-pot reaction approach reduced the risk of contamination as well as sample loss from multistep reaction. Indeed, it made the quantitative measurement more accurate [71].

- Microdroplets for RNA aptamers

In protocells systems, using droplets and heat enables mimic intercellular conditions that are fundamental to achieve pression over RNA aptamer, this method improves its microenvironment and consequently its folding and increases its in vivo biomolecule functionality [72].

## 6. Future Perspectives and Discussion

### 6.1. Comparative Performance of Microdroplet Gen Transfer Systems

Droplet-based microfluidics is a revolutionizing approach that provides a controllable microscale environment where different gene transfer mechanisms can be studied. In microbial systems such as *Streptococcus pneumoniae*, droplet confinement has enabled the direct observation of transformation events and macrorecombination at the precise moment of cell-to-cell interaction—phenomena previously associated with biofilms and in vivo conditions. These observations validate droplets as physiological environments that reveal the true magnitude and dynamics of genetic transfer in the pathogen.

In recalcitrant unicellular organisms such as microalga, microfluidic electroporation and mechanoporation platforms demonstrate clear performance benefits over bulk methods, especially in delivering foreign DNA. For instance, *Chlamydomonas reinhardtii* transformation via microfluidic electroporation achieved higher viability and produced stable, long-term transformants—an outcome that provides a powerful and versatile tool for both observing and manipulating genetic material.

Regarding transfection, microdroplet format enhances local concentrations and contact probability between cargo (nucleic acids, mRNA, protein or complexes) and the cell membrane; offering a controlled and homogeneous microenvironment that facilitates precise modulation of reaction kinetics. Therefore, exceeding plate-based controls efficiency as a microdroplet container works at cell resolution enabling stable genomic integration, scalability and automation.

In the realm of gene editing efficiency, a notable advance is the integration of droplet delivery with mechanoporation exemplified by the Droplet Cell Pincher (DCP). By producing transient perforations in both plasma and nuclear membranes, the DCP enables convective nuclear internalization of CRISPR-Cas9 RNPs and reports dramatic increases in editing efficiency (≈6.5× for single knockouts and ≈3.8× for double knockouts/HDR knock-ins relative to electroporation). The dual-membrane perforation allows the CRISPR-Cas9 machinery to be directly and rapidly forced into the nucleus, a process called convective internalization. Indeed, this technique is presented as a safer and more effective alternative to older methods. Its main advantage is its ability to bypass the nuclear envelope, a significant barrier that often limits gene-editing efficiency in methods relying on passive diffusion. By delivering the cargo directly into the nucleus.

In addition, ultrasonic levitation represents an emerging droplet-based technique that further expands the transfection tools. By maintaining cells and genetic cargo suspended within an acoustically stabilized droplet, this method enables reagent-free, contactless manipulation that minimizes surface adsorption and cross-contamination. Comparative studies using reporter genes such as Luciferase and Enhanced Green Fluorescent Protein (EGFP) demonstrated that transgene expression efficiency and overall cellular uptake were significantly higher in the ultrasonically levitated droplets compared to conventional tube-based transfections, even when assisted by mechanical shaking. Importantly, the data confirmed that the duration of ultrasonic levitation caused no significant damage to the plasmid DNA or the cell viability, underscoring that high efficiency and biocompatibility can be simultaneously achieved. Similarly, innovations in integrative approaches, such as TriDrop configuration (Figure 9), are elegant solutions designed to mitigate the harmful side effects typically associated with conventional bulk electroporation techniques, such as Joule heating, pH changes, and exposure to toxic electrolytic byproducts [50].

From an application perspective, microdroplets provide unique advantages in subsequent isolation and expansion of clones as they address the recovery of edited clones without antibiotic selection. Consequently, simplifying workflows and improving clonal fidelity. Indeed, precise derivation of isogenic cell lines is essential for regenerative medicine and disease modeling. In these biocompatible platforms, physical stress is reduced in contrast to traditional tools or robotic systems. A droplet containing a biological cell experiences a negligible electrical field and therefore their viability is maintained [70].

Notably, this approach provides more precise control over the environment and editing components while simultaneously reducing reagent consumption and processing time compared with traditional methods. Furthermore, the integration of emerging gene-editing systems such as CRISPR-Cas9 into the aqueous phase used for microdroplet generation, together with their combination with gene delivery methods such as viral vectors, electroporation, lipofection, ultrasound, or magnetofection, not only enhances transformation efficiency but also improves cell recovery and transgene expression [30,47,49,73].

In the context of cancer immunotherapy, one of the most promising technologies is the development of CAR-T cells. To date, the most widely used methods for their generation rely on viral vectors and electroporation, both of which involve high manufacturing costs. However, electroporation of T cells with naked mRNA through electroporation can reduce cell viability due to enzymatic degradation of the genetic material [74]. Interestingly, this limitation is considerably mitigated in microdroplet-based systems, which provide a controlled environment with minimal contamination [23,71].

As a cargo mRNA induces lower cellular cytotoxicity compared to nucleofection with pDNA [75]. Nevertheless, mRNA-based CAR-T cells generated by electroporation results in a short cytotoxic lifespan of only a few days [76]. Moreover, large-scale activation of T cells expressing CAR molecules can lead to tonic signaling and subsequent apoptosis, both undesirable for therapy [77]. Therefore, developing a system capable of providing controlled and uniform transfection of T cells could offer a viable path to overcome this limitation. One such example is microdroplet-based systems, which promote higher cell viability, achieving values above 80% in mRNA transfections and over 50% when plasmid DNA is used (Table 1).

As shown in Table 1, human primary lymphocytes T, K562, Hek293, Jurkat, and HeLa cells exhibited high transfection efficiencies, often higher than 80%, particularly when mRNA or CRISPR-Cas9 systems were used. In contrast, conventional methods show lower efficiencies in T-cell lines usually ranging from 45% to 70% when ribonucleoprotein systems are employed and around 50% when DNA is used [78,79]. Consistent with these observations, efficiencies of approximately 40% have been reported for K562 cells with iRNA [49], 70% for HEK293 cells when DNA is transferred [80], 63% for Jurkat cell with pDNA [81], and around 75% for HeLa cells when mRNA is used [82].

Although predominantly explored in the biomedical field, microdroplets-based systems have also expanded into other biotechnological applications. These include bioprocess engineering, where fungi transformation is optimized for be used to produce eukaryotic recombinant proteins; plant biotechnology, to improve the screening of transformed phenotypes; and clinical microbiology, to enhance donor–recipient cell interactions during the conjugation of non-domesticated bacterial strains. Collectively, these advances open new avenues for optimizing and standardizing high-yield biotechnological processes [62,63,64,67,68,69].

### 6.2. Emerging Trends (Materials and AI Integration)

We envision several routes for expanding gene-transfer in microdroplets and its applications.

- Materials to replace PDMS.

Although polydimethylsiloxane (PDMS) and soft-lithography have long served as the default for droplet microfluidics owing to their ease of prototyping [83,84], optical transparency, and facile gas permeability, their intrinsic limitations (such as absorption of small molecules, swelling in organic solvents, and variability in surface chemistry) increasingly drive researchers toward thermoplastic platforms for high-throughput and commercial deployment [85]. Thermoplastic polymers like Poly (methyl methacrylate) PMMA, Polyetherimide (PEI) [86], cyclic olefin copolymer (COC) which is a stiffer thermoplastic [87], COC/cyclic olefin polymer (COP), and polycarbonate lend themselves to direct micromachining (e.g., Computer numerical control (CNC) milling), hot embossing, and injection molding—all of which are compatible with scalable, reproducible manufacturing [88]. Furthermore, Si/glass platform is particularly well suited for pharmaceutical manufacturing because of its solvent compatibility and the ability to operate at high pressures [89]. That said, the shift is not trivial: challenges include achieving high-fidelity microfeature replication, reliable and biocompatible bonding strategies, and effective surface functionalization to preserve cell compatibility. Recent progress in thermoplastic bonding (e.g., low-temperature rapid bonding of PMMA) and hybrid prototyping approaches (e.g., combining 3D printed masters with CNC milled thermoplastic chips) shows promising momentum toward reducing development cycles without sacrificing manufacturability [88]. In the context of droplet microfluidics for eukaryotic gene editing, a pragmatic trajectory emerges we may retain PDMS during the early exploratory phase, but transition to thermoplastic or hybrid manufacturing for matured designs, to ensure device robustness, reagent compatibility, and cost-effective scale-up for translational applications.

- AI integration

At present, the concept of interdisciplinarity assumes an ever more significant position in modern research [90]. Modeling microfluidic devices is challenging due to complex nonlinear interactions, missing data for key parameters, and errors from manufacturing and operation. This means each passive droplet generation system is unique and requires specific adjustments to produce droplets of the desired size and rate. Consequently, a major goal in microfluidics is to create a universal method that can achieve target droplet characteristics across any device or platform [4]. Numerous investigations leveraging AI and machine learning (ML) methodologies have been conducted to forecast the optimal droplet dimensions for microfluidic devices. The findings revealed that the prediction precision surpassed 70% in most of the studies. This suggests that ML can be successfully applied to predict the useful droplet size. Dubey et al. created an automated droplet formation process utilizing an AI-driven mechanism for regulating droplet size. Each droplet produced by the microfluidic device is meticulously monitored via a vision system. The image processing unit functions to ascertain the droplet’s dimensions and provide feedback for the control system. Besides, they examined how variations in the continuous phase influence the same type of dispersed phase when the flow-rate ratio is modified by assessing 5 types of oils (sunflower oil, olive oil, rice bran oil, almond oil, mustard oil). The originality of this configuration resides in the creation of a droplet generation system that autonomously achieves the target droplet size without any human intervention. This research uses droplet dimensions data instead of direct images, which makes the algorithm immune to image noise and significantly faster to train. The study suggests that a deep neural network is the most effective method for predicting droplet sizes. Combining this AI approach with a rapid flow control system could lead to the creation of a fully automated microfluidic device [4].

In order to detect and quantify fluorescent droplets with a wide range of sizes, Song et al. implemented an advanced deep learning algorithm that executes semantic segmentation in conjunction with the circular Hough transform (CHT) to efficiently identify and quantify droplets from the low quality digital PCR imagery (e.g., images that are out of focus, as well as those exhibiting variations in morphology, dimensions, and fluorescence intensities) [91]. The integration of such artificial intelligence (AI)-based image analysis tools represents a major step forward for droplet-based transfection studies, as fluorescence intensity can serve as a direct proxy for transfection efficiency at the single-cell level. In this context, deep learning algorithms enable real-time classification of droplets containing highly fluorescent, successfully transfected cells, drastically reducing the need for manual inspection and post-processing. As these systems evolve, their coupling with automated droplet sorting and microfluidic control is expected to accelerate clone selection and data acquisition, ultimately reducing experimental time, reagent consumption, and variability across biological replicates. This convergence of microfluidics, fluorescence imaging, and AI-driven analytics will likely define the next generation of precision platforms for gene delivery and single-cell engineering.

In this context, DropAI generates picoliter-scale reactors and implements a fluorescent color-coding methodology to systematically address and screen extensive chemical combinations. The in-droplet screening process is enhanced through in silico optimization; wherein experimental findings are employed to train a machine-learning algorithm that estimates the contributions of various components and forecasts high-yield combinations. Through the application of DropAI, the authors have notably streamlined the composition of an *Escherichia coli*-based cell-free expression (CFE) system, realizing a fourfold decrease in the unit cost associated with the production of superfolder green fluorescent protein (sfGFP) [73]. Besides, the established *E. coli* model was successfully adapted to a *Bacillus subtilis*-based system through transfer learning [73].

Another parameter that AI could contribute to optimize is the applied voltage during electroporation in microdroplets. Electroporation-induced cellular rupture dynamics has been investigated on live breast cancer cells on a microfluidic electroporation chip array [92] but not in microdroplets yet. This has the potential to significantly impact gene therapy, regenerative medicine and disease modeling.

## 7. Conclusions

Overall, droplet-based microfluidics offers significant advantages over traditional methods. It enables more precise control over the cellular environment as it increases contact probability between cargo and cell membrane. Besides, it reduces reagent consumption and processing time, and improves cell viability. In addition, gene transfer performed in microdroplets provides an accurate modulation of reaction kinetics. Accordingly, microdroplet based microfluidics has improved transfection efficiency of hard-to-transfect cells, high throughput, and scalability. Furthermore, it offers a novel approach for the introduction of genome-editing agents into single cells enabling stable genomic integration. Moreover, microdroplets facilitate sorting, recovery and expansion of engineered clones which are essential for regenerative medicine and disease modeling.

In the context of eukaryotic cell transfection, recent advances highlight that microdroplet confinement enhances delivery efficiency and reproducibility by minimizing cell-to-cell variability and providing a finely tuned microenvironment for gene uptake. This confined setting promotes higher integration rates in transposase-mediated delivery and supports precise clonal recovery following CRISPR/Cas9 editing. Together, these developments position droplet-based systems as a cornerstone for next-generation platforms in gene editing and stem cell engineering.

## Figures and Tables

**Table 1:** Microdroplet based gene delivery system performance metrics.

**Part A. Transformation**						
**Biological Sample**	**Target Cargo**	**Concentration/Size**	**Viability (%)**	**Efficiency (%) or Ratio ^1^**	**Throughput**	**Ref.**
*C. reinhardtii*	DNA fragments	40 µg/mL	81	0.081, ratio: 8.14 ± 0.20 × 10^−4^	-	[20]
*C. reinhardtii*	pDNA	1 µg/mL	80–90	20.7 ± 6.2	High	[47]
*C. reinhardtii*	pDNA	8104 bp	>80	≈10–20 (1–5% stable transformation)	High	[48]
*E. coli*	pDNA	10 pmol/µL	-	98 ± 3	High	[22]
*S. pneumoniae* (CP2204 recipient + CP2215 donor)	Native chromosomal DNA	-	40 (Donor) 60 (Recipient)	-	≈50%	[35]
*S. pneumoniae* (CP2204 recipient + CP2215 donor)	Genomic DNA	5 µg/mL	-	-	≈50%	[37]
**Part B. Transfection**						
**Biological Sample**	**Target Cargo**	**Concentration/Size**	**Viability (%)**	**Efficiency (%)**	**Throughput**	**Ref.**
CHO cells	pDNA	100 µg/mL	≈68	≈11	-	[19]
K562	mRNA	20 µg/mL	>80	≈100	High	[62]
Human primary T lymphocytes	mRNA	20 µg/mL	>82	≈90	High	
K562	pDNA	7.9 kbp	≈80	45%	High	
K562	mRNA	20 µg/mL		>98	1 × 10^6^ cells/mL	[64]
H1299	pDNA	10.5 kbb	-	≈70%	-	[21]
SMMC-7721	pNOS	25 ug/mL	51	61.5	-	[30]
Jurkart cells K562 HEK-293T NK-92			>75	>90	6 × 10^7^ cells/mL	[31]
HEK293	mRNA	2 pg/cell	>90	>90	High	[50]
	pDNA	≈5 kb	90	71		
HeLa	pDNA	≈5 kb	>90	≈60		
HEK293T	pDNA	5 ng/µL	>80	60	High	[27]
MCF7	pDNA	20 µg/mL	>80	40–60%	High	
hiPSC	pDNA-CAG-mCerulean + PiggyBac	2.5–10 µg/mL	>85	70	High	[9]
hiPSC	CRISPR/Cas9 pDNA	10 µg/mL	>80	94–96	High	[38]
K562	CRISPR-Cas9/mRNA y pDNA	100 µg/mL	>75	>62	High	[38]
Huh-7	pDNA	60 µg/mL	-	Qualitative	Qualitative	[49]
Primary human T cells CART	mRNA	50 µg/mL	-	>60 (2× to conventional technics)	-	[52]
HeLa cells	pDNA tdTomato and mVenus	100 ng/μL	-	90 Co-transfection ≈ 100	High	[51]
HeLa cells	pDNA	5 ng/μL and 30 ng/μL	-	≈100	High	[52]

^1^ number of positive colonies/total number of cells used in electroporation.

## Data Availability

No new data were created or analyzed in this study. Data sharing is not applicable to this article.

## Abbreviations

ASO	antisense oligonucleotides
CAR	chimeric antigen receptor
Cas9	CRISPR-associated protein 9
CFE	cell-free expression
CHO	Chinese hamster ovary
CHT	circular Hough transform
CNC	Computer Numerical Control
COC	Cyclic Olefin Copolymer
COP	Cyclic Olefin Polymer
CRISPR	Clustered regularly interspaced short palindromic repeats
DCP	Droplet Cell Pincher
DMF	Digital Microfluidics
EGFP	Enhanced Green Fluorescent Protein
EWOD	electrowetting-on-dielectric
HDR	homology-directed repair
hiPSCs	human induced pluripotent stem cells
miRNA	micro RNA
pDNA	plasmid DNA
PMMA	Poly(methyl methacrylate)
RNP	ribonucleoprotein
SCTTs	Single-Cell Transfection Technologies
sfGFP	superfolder green fluorescent protein
SG3	Sustainable Goal 3
shRNA	short hairpin RNA
siRNA	small interfering RNA
TR	transfection reagent
